# Chromosomal Localization of Candidate Genes for Fiber Growth and Color in Alpaca (*Vicugna pacos*)

**DOI:** 10.3389/fgene.2019.00583

**Published:** 2019-06-19

**Authors:** Mayra N. Mendoza, Terje Raudsepp, Fahad Alshanbari, Gustavo Gutiérrez, F. Abel Ponce de León

**Affiliations:** ^1^Programa de Mejoramiento Animal, Universidad Nacional Agraria La Molina, Lima, Peru; ^2^Molecular Cytogenetics and Genomics Laboratory, Texas A&M University, College Station, TX, United States; ^3^Department of Animal Science, University of Minnesota, Minneapolis, MN, United States

**Keywords:** alpaca, chromosomes, FISH, mapping, fiber, color, genes

## Abstract

The alpaca (*Vicugna pacos*) is an economically important and cultural signature species in Peru. Thus, molecular genomic information about the genes underlying the traits of interest, such as fiber properties and color, is critical for improved breeding and management schemes. Current knowledge about the alpaca genome, particularly the chromosomal location of such genes of interest is limited and lags far behind other livestock species. The main objective of this work was to localize alpaca candidate genes for fiber growth and color using fluorescence *in situ* hybridization (FISH). We report the mapping of candidate genes for fiber growth *COL1A1*, *CTNNB1*, *DAB2IP*, *KRT15*, *KRTAP13-1*, and *TNFSF12* to chromosomes 16, 17, 4, 16, 1, and 16, respectively. Likewise, we report the mapping of candidate genes for fiber color *ALX3*, *NCOA6*, *SOX9*, *ZIC1*, and *ZIC5* to chromosomes 9, 19, 16, 1, and 14, respectively. In addition, since *KRT15* clusters with five other keratin genes (*KRT31*, *KRT13*, *KRT9*, *KRT14*, and *KRT16*) in scaffold 450 (Vic.Pac 2.0.2), the entire gene cluster was assigned to chromosome 16. Similarly, mapping *NCOA6* to chromosome 19, anchored scaffold 34 with 8 genes, viz., *RALY*, *EIF2S2*, *XPOTP1*, *ASIP*, *AHCY*, *ITCH*, *PIGU*, and *GGT7* to chromosome 19. These results are concordant with known conserved synteny blocks between camelids and humans, cattle and pigs.

## Introduction

The alpaca (*Vicugna pacos*) is a domesticated South American camelid adapted to the Andean climate conditions. They are economically important in Peru as a fiber production species benefiting the small shareholders living in this geographical region (Quispe et al., 2009). Alpaca fiber is highly valued in the international market because of its softness and resistance (Crispín, 2008). Alpacas carry a cultural value because of their historical importance, millenary tradition, ancestral Peruvian identity and unique characteristics derived from their adaptation to the Andean geography and climate (Yucra, 2017). Alpaca meat is highly valued for its high protein and low cholesterol content (Hack, 2001), and continues serving rural population of Altiplano as an important source of protein (Cruz et al., 2017).

Management systems promoting the improvement of alpaca herd productivity have not yet been adopted widely (Quispe et al., 2009). Actual research is orientated to the application of genetic improvement technologies that would decrease fiber diameter, increase fleece weight, and establish uniform color herds (Morante et al., 2009). Genomic selection using single nucleotide polymorphisms (SNPs)-based genotype-phenotype associations, offers the best option presently available. To apply genomic selection in alpacas, it is necessary to identify and map SNPs throughout the genome and associate them with genes that control economic productive traits. In turn, mapping candidate genes already reported in association to color and fiber characteristics, as well as SNPs, will contribute to understanding the organization of the alpaca genome and genome-wide selection of appropriate markers to develop molecular marker microarrays.

Cytogenetic analysis has demonstrated that all camelids share the same chromosome number (2n = 74) with essentially similar chromosome morphology and banding patterns (Hsu and Benirschke, 1967; Taylor et al., 1968; Bianchi et al., 1986). The first camelid chromosome map was based on Zoo-FISH revealing evolutionarily conserved synteny segments across the dromedary, human, cattle and pig (Balmus et al., 2007). This information was instrumental for starting systematic gene mapping in these species and the first cytogenetics maps for the alpaca genome were developed only recently (Avila et al., 2014a,b, 2015). Because of difficulties to unambiguously identify camelid chromosomes (Di Berardino et al., 2006; Avila et al., 2014b), the 230 cytogenetically mapped markers in alpaca (Avila et al., 2014a) will serve as critical references for FISH-mapping new genes and markers.

The aim of this study was to cytogenetically map 11 alpaca candidate genes for fiber growth and coat color to progress the development of alpaca cytogenetic map and chromosomal anchoring the reference sequence.

## Materials and Methods

### Chromosome Preparations

Alpaca chromosome slides were prepared from peripheral blood lymphocytes of normal alpacas according to standard protocols (Raudsepp and Chowdhary, 2008). We used Concanavalin A (Con A from *Canavalia ensiformis*, 20 μg/ml; Sigma Aldrich) as the mitogen, instead of Pokeweed, because Con A stimulates better proliferation of alpaca blood lymphocytes (Avila et al., 2015).

### Gene Selection and Primer Design

Genes for cytogenetic mapping were retrieved from publications. Candidate genes regulating fiber growth characteristics, *COL1A1*, *CTNNB1*, *DAB2IP*, *KRT15*, and *TNFSF12* (Fernandez, 2015), and *KRTAP13-1* (Florez, 2016); candidate genes that regulate the expression of fiber color, *NCOA6-agouti* chimera (Chandramohan et al., 2013); *ZIC1*, *ZIC5*, and *SOX9* which conform the neural crest gene regulatory network (Simoes-Costa and Bronner, 2013), and the *ALX3* transcription factor that regulates melanocyte differentiation in striped rodents (Cuthill et al., 2017). Gene specific sequences were retrieved from VicPac 2.0.2 (GCA_000164845.3) at the NCBI (National Center for Biotechnology Information). Since each of the selected genes are members of gene super-families, sequences that characterized these super-families were identified using the BLASTp^1^ and Spling^2^ tools and manually removed from each gene FASTA sequence. This way unique sequences for each specific gene were obtained. The gene sequences were masked for repeats in RepeatMasker^3^. Gene-specific PCR primers were designed with Primer3 (Untergasser et al., 2012)^4^ and Primer-BLAST^5^ software packages. The primers were tested by *in silico* PCR^6^ and optimized on alpaca genomic DNA.

Overgo primers were designed manually from 36 to 52 bp size sequence within the PCR amplicon. We designed a 24 bp forward primer from the first nucleotide at the 5′ end position of the selected region. The reverse primer was designed starting at the 3′ end of the selected region, ending with 8 nucleotides overlapping the forward primer. The overlapping section and the single strand sections of the forward and reverse primers, contained 50–60 (±5) % GC (we used GC calculator^7^). PCR and overgo primers for each gene are presented in Table 1.

**Table 1:** Gene specific PCR and overgo primers.

	Identified		PCR product
Gene symbol	BAC clones	PCR primer 5′-3′	size (bp)	Overgo primer sequence 5′-3′
*ALX3*	**115I10**	F: TATGTCTCCGTACTCCCACTCTCR: GGAGACTTATAGTCGTCATCTGG	161	F: GCTCTAGGGGGCCACAGCTTTGAGR: CGTCATCTGGGGAGGGCTCAAAGC
		R: GGAGACTTATAGTCGTCATCTGG		R: CGTCATCTGGGGAGGGCTCAAAGC
*COL1A1*	198E13	F: CCATTGGTAGTGTTGGTGCT	365	F: GCCCTGTTGGCAAAGAAGGCAGCA
	**204B18**	R: AGGGAAGCCTCTTTCTCCTC		R: TCACCACGAGGACCTTTGCTGCCT
	264O11			
	271L20			
	295O17			
*CTNNB1*	129B09	F: ATCCCAGCTATCGTTCTTTTCA	300	F: CACTCCGGTGGATACGGACAGGAT
	**150A21**	R: CCTACCAACCCAAGTCTTTCTG		R: GGTCCATACCCAAGGCATCCTGTC
*DAB2IP*	**101B06**	F: TACTGAGAACGGCGAGTTCA	107	F: GAACGGCGAGTTCAGAAACAGCAGCAA
		R: AAAGCTCAGCCTCTCTCTCG		R: CGTGCCTGGGACACTTGAATTGCTGCT
*KRT15*	263E22	F: GGCAAAGTCCGCATCAATGTT	218	F: TGGCCAGAGGGGCCAGAAGGGCAAA
	**268A9**	R: ATGCCAAGCAGCCAACTAGG		R: CCCCTCTGGGTCTAGAGTTTGCCCT
	274A22			
*KRTAP13-1*	336H05	F: GCAAAGGCTACTTCCTGGTCTA	109	F: TCCAGAAGCTGTGGGTCCAGTGG
	**368J2**	R: ATTGGATGGCAGGATCCACAG		R: TCCAGAACCCAGAGATCCACTGGA
	408J12			
	413H10			
*NCOA6*	34F15	F: CCCAAGATTTTCTAAAGACAGGAA	151	F: CAGCTGTGTTTACAACTCCTCCAGCCAAG
	46J23	R: CTGGTCAGTATGGGCTTATCTCTT		R: CTGGTCAGTATGGGCTTATCTCTTGGCTG
	**59N23**			
	86O24			
*SOX9*	13O23	F: AAATGCTCTTATTTTTCCAACAGC	220	F: GTGTTATGGGATCAGTTTGGGGGGTTA
	30B6	R: AATCACAAAGCCTGAGGAATTAAG		R: CTGAGGAATTAAGCAAAGCTAACCCCC
	32I21			
	58P4			
	68P18			
	115A15			
	122H18			
	169O5			
	172F10			
	186L14			
	202F10			
	**231H17**			
	249C14			
	279H10			
	297J24			
	306O5			
*TNFSF12*	169O5	F: GACCTGAATCCCCAGACAGA	94	F: AGCCAGGACACCGTGTCTTTCCTG
	133N9	R: GTGGTTTCCGGCCTTTAGGT		R: GAGGCCGAACCAGTTTCAGGAAAG
	**172F10**			
*ZIC1*	127I17	F: AGTCCGCGTTCAGAGCACTAT	192	F: GCGCCGGCGCTTTCTTCCGCTACATG
	**135I16**	R: GAAAGTTTTGTTGCACGACTTTTT		R: CTGTTTGATGGGCTGGCGCATGTAGC
*ZIC5*	211H22	F: GCAAACTTTCTGCAAGTGCAAC	199	F: AGGGGGCACGAAGCGAAAGCGAAG
	**224A3**	R: GGAAGCCTGTCATATTCTGAAAC		R: CTGTGCTCACTGACGCCTTCGCTT

*BAC numbers in bold denote those that were used for gene chromosomal localization by FISH*.

### Alpaca CHORI-246 Library Screening and BAC DNA Isolation

BAC clones containing sequences of the selected genes were identified as described by Avila et al. (2014b). Briefly, pools of radioactively labeled [(^32^P) dATP/dCTP] overgo primers were hybridized to CHORI-246 alpaca BAC library^8^ filters. Filters were exposed to autoradiography films and positive BAC clones were identified and picked from the library. BACs corresponding to individual genes were identified by PCR with gene-specific primers. BAC DNA was isolated with the Plasmid Midi Kit (Qiagen) and evaluated for quality by electrophoresis in 1% agarose gels.

### Probe Labeling, FISH and Microscopy

BAC DNA labeling, hybridizations and signal detection were carried out according to standard protocols (Raudsepp and Chowdhary, 2008). The DNA of individual BACs was labeled with biotin or digoxigenin using DIG- or Biotin-Nick Translation Mix (Roche Diagnostics) and the manufacturer’s protocol. Because the known difficulties to unambiguously identify camelid chromosomes, we consulted Zoo-FISH data (Balmus et al., 2007) and the 230-marker cytogenetic map (Avila et al., 2014a) to infer the most probable chromosome location for each candidate gene. Based on these predictions, BACs containing new genes were co-hybridized with a differently labeled reference gene from the cytogenetic map (Table 2). Biotin- and dig-labeled probes were detected with avidin-FITC (Vector Laboratories) and anti-dig-rhodamine (Roche Applied Science), respectively. Chromosomes were counterstained with 4′,6-diamidino-2-phenylindole (DAPI) and identified according to the nomenclature proposed by Balmus et al. (2007) and Avila et al. (2014b). Images were captured and analyzed using a Zeiss Axioplan 2 fluorescence microscope, equipped with the Isis Version 5.2 (MetaSystems GmbH) software. At least 10 images were captured and analyzed for each experiment.

**Table 2:** Summary data of newly mapped genes and reference markers (Avila et al., 2014b).

	Gene					Reference marker		
			Chromosomal	VicPac2.0.2	CHORI 246		CHORI 246
	Symbol	Name	location	scaffold	BAC Clone	Name	BAC Clone	Location
Fiber growth candidate genes	*COL1A1*	Collagen type I alpha 1 chain	16q13	**377**	204B18	*DDX52*	18J7	16p14prox
	*CTNNB1*	Catenin beta 1	17q12-q13	23	150A21	*MITF*	33H2	17q14
	*DAB2IP*	Disabled homolog 2-interacting protein	4q34	52	101B6	*GG_478*	71E21	4q34
	*KRT15*	Keratin 15	16q12-q13	**450**	268A9	*AP2B1*	156N10	16p13
	*KRTAP13-1*	Keratin Associated Protein 13-1 Like	1q33	101	368J2	*SOX2*	24K2	1q19
	*TNFSF12*	TNF superfamily member 12	16p13	**387**	172F10	*KCNJ16*	408P6	16q16
Fiber color candidate genes	*NCOA6*	Nuclear receptor coactivator 6	19q12	34	59N23	*ASIP*	18C13	19q12
						*BMP7*	93P6	19q22
	*ZIC1*	Zinc finger protein ZIC 1	1q13-q14	**35**	135I16	*SOX2*	24K2	1q18-q21
	*ZIC5*	Zic family member 5	14q15-q16	**84**	224A3	*RB1*	89N13	14p13
	*SOX9*	SRY-box 9	16q17	15	231H17	*KCNJ16*	408P6	16q16
	ALX3	ALX homeobox 3	9q24-q25	4	115I10	*GG1068*	2N23	9q14

*VicPac2.0.2 scaffolds in bold denote those that were chromosomally assigned first time in this study*.

## Results

**Figure 1:**
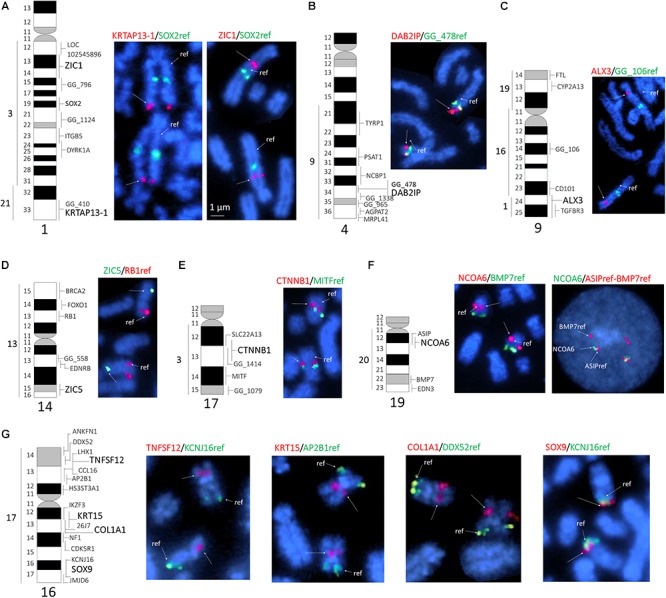
FISH mapping of selected genes to alpaca chromosomes. **(A)** *ZIC1* and *KRTAP13-1* to chr1; **(B)** *DAB2IP* to chr2; **(C)** *ALX3* to chr9; **(D)** *ZIC5* to chr14; **(E)** *CTNNB1* to chr17; **(F)** *NCOA6* to chr16, and **(G)** *TNFSF12*, *KRT15*, *COL1A1*, and *SOX9* to chr16. The left side of each sub-figure includes a G-banded ideogram showing conserved synteny with human chromosomes (far left) and all cytogenetically mapped markers (right). The markers mapped in this study are in larger and bold font. The right side of each sub-figure shows partial metaphase spreads with dual-color FISH results. The newly mapped genes are denoted with arrows; reference genes for chromosome identification are denoted with arrows and “ref.” The symbols of newly mapped and reference genes are shown above each figure in green or red font colors, which match with the colors of FISH signals on chromosomes. In **(F)**, we show refined ordering of *NCOA6* in relation to *ASIP* in interphase chromosomes (far right) using *BMP7* is an anchoring marker; the order is cen-*ASIP*-*NCOA6*-*BMP7*-tel.

Altogether, we identified 41 BAC clones that collectively contained the 11 genes of interest. Clones for individual genes were identified by PCR with gene-specific primers (Table 1), and one clone per each gene was selected for FISH mapping. In this manner, we assigned 11 BAC clones to eight different alpaca autosomes. Most of the candidate genes were mapped to a specific G-band or a range of G-bands (Table 2). Previously mapped reference markers (Avila et al., 2014b) confirmed chromosome identification and helped to position new genes in the centromere-telomere field (Figure 1). Four genes were located in chromosome 16 (chr16), and 2 genes in chr1, whereas the remaining five genes mapped to five different chromosomes (Figure 1 and Table 2). In chr19, the *NCOA6* gene overlapped with *ASIP* in 19q12, and their relative order was resolved by interphase FISH using *BMP7* as the second reference marker. The order of the three genes was revealed as cen-*ASIP*-*NCOA6*-*BMP7*-tel (Figure 1F, far right). Location of *CTNNB1*, *DAB2IP*, and *SOX9* in chr17, chr4 and chr16, respectively, was confirmed by co-hybridized reference markers. No genes were assigned to chromosome arms that previously did not have a mapped marker. No discrepancies of the known conserved synteny blocks between camelids, cattle and human (Balmus et al., 2007) were observed.

## Discussion

We report the cytogenetic mapping of 11 new genes in the alpaca genome, which together with prior FISH maps (Avila et al., 2014a,b) takes the tally of all chromosomally mapped markers for this species to 241. This is not a high number but an important step forward for the development of chromosomally anchored reference genomes for the alpaca and other camelids. Furthermore, among the 11 markers mapped in this study, five belong to VicPac2.0.2 scaffolds that were not represented in previous maps (Avila et al., 2014b). This implies that the entire scaffold 35, anchored by *ZIC1*, maps to chr1; scaffold 84, anchored by *ZIC5*, maps to chr14, and scaffolds 377, 387, and 450, anchored by *COL1A1*, *TNFSF12*, and *KRT15*, respectively, map to chr16 (Table 2).

As the goal of this study was cytogenetic mapping of candidate genes related to fiber growth and color synthesis, we bioinformatically inspected all VicPac2.0.2 scaffolds containing the 11 mapped markers (Table 2) for additional genes of interest. In scaffold 450 (331,325 bp, NW_005883152.1), which was newly assigned to chr16q12-q13 by FISH mapping *KRT15* (Figure 1G), there is a tandemly arranged cluster of five more keratin genes around *KRT15*, viz., 5′- *KRT31*- *KRT15* -*KRT13*- *KRT9*- *KRT14*-*KRT16 – 3*′ (Fernández et al., 2019). Thus, our results allow the assignment of five more keratin genes to chr16q12-q13 (Table 3). This makes alpaca chr16 as a main target for identifying sequence variants potentially associated with hair texture and growth because three of the six candidate genes for fiber growth characteristics (Table 2), viz., *KRT15* with the keratin cluster, *COL1A1*, and *TNFSF12* (Fernandez, 2015), map to this chromosome. This also implies that phenotypic characteristics determined by these genes may show particular inheritance patterns due to close linkage. Among the many known molecular components of the mammalian hair follicle (Rompolas and Greco, 2013), keratins and collagens are perhaps most studied (Toivola et al., 2015) and associated with various hair texture characteristics in several mammalian species including humans (Shimomura et al., 2010), dogs (Cadieu et al., 2009), horses (Balmer et al., 2017; Morgenthaler et al., 2017) and alpacas (Fan et al., 2011). Here we considered as candidate genes for alpaca hair texture also genes related to apoptosis regulation and formation of the hair follicle, such as *CTNNB1*, *TNFSF12*, and *DAB2IP*. *TNFSF12* and *DAB2IP* have roles in WNT/β-catenin signaling system (Xie et al., 2010), which controls hair follicle morphogenesis and stem cell differentiation in the skin (Huelsken et al., 2001). SNP variants in these genes have been associated with traits of interest (Farhadian et al., 2018) and used for genomic selection programs in sheep, goat (Rupp et al., 2016) and cattle (Wiggans et al., 2017).

**Table 3:** Summary data of the genes positionally associated with the genes mapped in this study.

				Inferred VPA
Scaffold				chromosomal
VicPac2.0.2	Mapped marker	Positionally associated markers		location
		Gene symbol	Gene name	
450	*KRT15*	*KRT31*	Keratin, type I cuticular Ha1	16q12-q13
		*KRT13*	Keratin, type I cytoskeletal 13	16q12-q13
		*KRT9*	Keratin 9	16q12-q13
		*KRT14*	Keratin 14	16q12-q13
		*KRT16*	Keratin, type I cytoskeletal 16	16q12-q13
34	*NCOA6*	*RALY*	RALY heterogeneous nuclear ribonucleoprotein	19q12
		*EIF2S2*	Eukaryotic translation initiation factor 2 subunit beta	19q12
		*XPOTP1*	Exportin for tRNA pseudogene 1	19q12
		*AHCY*	Adenosylhomocysteinase	19q12
		*ITCH*	Itchy E3 ubiquitin protein ligase	19q12
		*PIGU*	Phosphatidylinositol glycan anchor biosynthesis class U	19q12
		*GGT7*	Gamma-glutamyltransferase 7	19q12

Therefore, microsatellites that have been identified in the alpaca *COL1A1*, *TNFSF12*, and *DAB2IP* (Fernandez, 2015) are potential polymorphic markers for selection in this species.

Among the candidate genes for hair color, mapping *NCOA6* to chr19q12 was of particular interest because it anchored a closely linked group of several other potential coat color genes from scaffold 34 (12,494,946 bp, NW_005882736.1) to this chromosome (Table 3). The closely linked gene cluster comprises *RALY*, *EIF2S2*, *XPOTP1*, *ASIP*, *AHCY*, *ITCH*, *PIGU*, *NCOA6*, and *GGT7*, of which only *ASIP* has been previously mapped (Avila et al., 2014b). In this study, we showed that *NCOA6* is overlapping with *ASIP* in chr19q12 (Figure 1F) which is consistent with the known organization of the agouti locus in alpacas, where the 5′UTR of the *ASIP* gene contains 142 bp of the *NCOA6* gene sequence (Chandramohan et al., 2013). The role of *ASIP* in regulation of pigment production in mammals is well established (Suzuki, 2013). Mutations in this gene have shown to cause the black coat color phenotype in different species, such as guinea pigs (Lai et al., 2019), black-bone chicken (Yu et al., 2019), sheep (Norris and Whan, 2008; Royo et al., 2008), Iranian Markhoz goats (Nazari-Ghadikolaei et al., 2018), donkeys (Abitbol et al., 2015), horses (Rieder et al., 2001), dogs (Kerns et al., 2004), cats (Eizirik et al., 2003), and impala antelope (Miller et al., 2016). In camelids the agouti signaling protein gene (*ASIP*) is involved in fiber color development in alpacas (Bathrachalam et al., 2011; Chandramohan et al., 2013), llamas (Daverio et al., 2016) and dromedaries (Almathen et al., 2018; Alshanbari et al., 2019). Sequence variants (SNPs) in other genes from this linkage group have been associated with color phenotypes in several mammalian species. For example, coat color of the Nanjiang Yellow goat has been associated with SNPs in the *RALY-EIF2S2* locus (Guo et al., 2018), tandem duplication encompassing *ASIP* and *AHCY* coding regions and the *ITCH* promoter region have been reported as the genetic cause of the dominant white coat color of white/tan (*A^Wt^*) *agouti* sheep (Norris and Whan, 2008), and *RALY*, *ASIP*, *AHCY*, and *ITCH* are associated with brown and black color coat in Iranian Markohz goat (Nazari-Ghadikolaei et al., 2018). Melanocytes, the cells that are responsible for skin pigmentation, are derived from neural crest cells from all axial levels (Betancur et al., 2010). Therefore, genes involved in neural crest generation, such as *ZIC* genes (Aruga, 2004), are potential candidates for fiber color development. Likewise, *SOX9* is involved in the differentiation of neural crest cells into chondrocytes (Simões-Costa and Bronner, 2015) and cooperates with other cofactors in chondrocytes to regulate expression of *COL2A1* in humans (Hattori et al., 2008). Furthermore, *SOX9* is a key player in ultraviolet B radiation-induced melanocyte differentiation and pigmentation by directly regulating *MITF* (Passeron et al., 2007). *MITF* is involved in melanogenesis regulation in alpaca (Wang et al., 2017) and plays a role in the production of white coat color in the llama (Anello et al., 2019). Finally, *ALX3* is involved in color differentiation in striped rodents (Cuthill et al., 2017), and proposed as a target melanoma gene fusion in humans (Berger et al., 2010). Also, Marín et al. (2018) used the genetic variation of *MC1R* and *ASIP* genes, that control coat color, to differentiate between wild and domestic South American camelids.

In summary, the findings of this study facilitate the improvement and chromosomal assignment of the alpaca genome reference sequence. This, in turn, is critical for correct assembly of newly sequenced individual animals and the discovery of sequence variants in candidate genes for fiber characteristics, coat color and other traits of interest. For instance, Alshanbari et al. (2019) have recently assign the *MC1R* gene to camelid chr21 that is not in line with the human-camelids Zoo-FISH synteny map. In addition, improving the alpaca cytogenetic map provides new molecular markers for clinical cytogenetics in alpacas and other camelids, thus facilitating chromosome identification in these complex karyotypes. Finally, cytogenetic mapping of specific genes refines the Zoo-FISH information (Balmus et al., 2007), reveals new evolutionary conserved synteny segments between camelids and other mammals, and adds to our knowledge about camelid chromosome evolution.

## Ethics Statement

The cell cultures were prepared from alpaca blood samples obtained in accordance with the United States Government Principles for the Utilization and Care of Vertebrate Animals Used in Testing, Research and Training, approved by Animal Use Protocol AUP #2011-96, # 2018-0342 CA and CRRC #09-47 at Texas A&M University.

## Author Contributions

FPdL and GG conceived and supervised the study. MM conducted the experimental work. FA contributed to the BAC screening. MM and TR analyzed the data. MM wrote the manuscript in close consultation with FPdL, TR, and GG. All authors read and approved the final version of the manuscript.

## Conflict of Interest Statement

The authors declare that the research was conducted in the absence of any commercial or financial relationships that could be construed as a potential conflict of interest.

## Footnotes

1 https://blast.ncbi.nlm.nih.gov/Blast.cgi?PAGE=Proteins

2 https://www.ncbi.nlm.nih.gov/sutils/splign/splign.cgi?textpage=online&level=form

3 http://www.repeatmasker.org/

4 http://bioinfo.ut.ee/primer3-0.4.0/primer3/

5 https://www.ncbi.nlm.nih.gov/tools/primer-blast/index.cgi?LINK_LOC=BlastHome

6 https://genome.ucsc.edu/cgi-bin/hgPcr

7 http://www.sciencelauncher.com/oligocalc.html

8 https://bacpacresources.org/libraries.htm
